# Knockdown of Salusin-*β* Improves Cardiovascular Function in Myocardial Infarction-Induced Chronic Heart Failure Rats

**DOI:** 10.1155/2021/8896226

**Published:** 2021-08-10

**Authors:** Yu Xu, Yan Pan, Xingxing Wang, Aidong Chen, Xinyu Tang, Xuanxuan Liu, Ying Han

**Affiliations:** ^1^Key Laboratory of Targeted Intervention of Cardiovascular Disease, Collaborative Innovation Center of Translational Medicine for Cardiovascular Disease, Nanjing Medical University, Nanjing, Jiangsu 211166, China; ^2^Department of Physiology, Nanjing Medical University, Nanjing, Jiangsu 211166, China; ^3^Department of Physiology and Pathologic Physiology, Kangda College of Nanjing Medical University, Lianyungang, Jiangsu 222000, China

## Abstract

Salusin-*β* is a biologically active peptide with 20 amino acids that exerts several cardiovascular activity-regulating effects, such as regulating vascular endothelial function and the proliferation of vascular smooth muscle cells. However, the regulatory effects of salusin-*β* in myocardial infarction-induced chronic heart failure (CHF) are still unknown. The current study is aimed at investigating the effects of silencing salusin-*β* on endothelial function, cardiac function, vascular and myocardial remodeling, and its underlying signaling pathways in CHF rats induced by coronary artery ligation. CHF and sham-operated (Sham) rats were subjected to tail vein injection of adenoviral vectors encoding salusin-*β* shRNA or a control-shRNA. The coronary artery (CA), pulmonary artery (PA), and mesenteric artery (MA) were isolated from rats, and isometric tension measurements of arteries were performed. Compared with Sham rats, the plasma salusin-*β*, leptin and visfatin levels and the salusin-*β* protein expression levels of CA, PA, and MA were increased, while the acetylcholine- (ACh-) induced endothelium-dependent vascular relaxation of CA, PA, and MA was attenuated significantly in CHF rats and was improved significantly by salusin-*β* gene knockdown. Salusin-*β* knockdown also improved cardiac function and vascular and myocardial remodeling, increased endothelial nitric oxide synthase (eNOS) activity and nitric oxide (NO) levels, and decreased NAD(P)H oxidase activity, NOX-2 and NOX-4 expression, and reactive oxygen species (ROS) levels in arteries in CHF rats. The effects of salusin-*β* knockdown in CHF rats were attenuated significantly by pretreatment with the NOS inhibitor L-NAME. These results indicate that silencing salusin-*β* contributes to the improvement of endothelial function, cardiac function, and cardiovascular remodeling in CHF by inhibiting NAD(P)H oxidase-ROS generation and activating eNOS-NO production.

## 1. Introduction

Chronic heart failure (CHF) is the terminal stage of a variety of cardiovascular diseases and is characterized by progressive left ventricular dysfunction and declining ejection fraction (EF), usually accompanied by vascular endothelial dysfunction, neuroendocrine activation, and ventricular remodeling [1–3]. Among them, vascular endothelial dysfunction usually occurs at the early stages of heart failure and is closely related to the development of CHF and is also a predictor of adverse events, such as cardiovascular remodeling in CHF patients [4, 5]. Normally, vascular endothelial cells (VECs) can release vasoconstrictor factors, such as endothelin-1, and vasodilatation factors, such as nitric oxide (NO), endothelium-derived hyperpolarizing factor (EDHF), and prostaglandin (PGI2), to control vascular tone. When endothelial dysfunction occurs, the release of vasoconstrictor factors and vasodilatation factors from VECs becomes imbalanced. Among these, the decrease in NO bioavailability plays the most important role in CHF [6–8], which results in the decline of vascular relaxation and the increase in vascular contraction function [9, 10]. Attenuated vascular relaxation caused by endothelial dysfunction in the coronary artery (CA) in CHF decreases vascular compliance, reduces the blood supply to the heart, and subsequently causes myocardial ischemia and angina and further deterioration of cardiac function [11, 12]. Attenuated pulmonary artery (PA) relaxation in CHF increases pulmonary vascular resistance and might contribute to the high incidence of pulmonary hypertension due to left heart disease [13, 14]. Due to the enormous quantity of mesenteric artery (MA), attenuated MA relaxation increases peripheral resistance and cardiac afterload, leading to further cardiac dysfunction. It is necessary to find therapeutic methods or interventions to correct the endothelial dysfunction of heart failure and then improve cardiac function and prevent the progression of CHF. Acetylcholine (ACh) stimulates VECs to release NO and induces vascular smooth muscle cells (VSMCs) relaxation. Leptin and visfatin, which are involved in regulating endothelial function and NO-dependent vasorelaxation, have been proposed as clinical markers of endothelial dysfunction and vascular injury in cardiovascular diseases [15–17]. Therefore, measurements of ACh-induced vasodilatation and plasma leptin and visfatin levels are usually used to evaluate endothelial function.

The salusin family includes salusin-*α* and salusin-*β*, which are translated from the alternative spliced mRNA of torsin family 2 member A (TOR2A) [18–20]. Salusin-*β* is an endogenous biologically active polypeptide with 20 amino acids, and salusin-*α* has 28 amino acids. Compared to salusin-*α*, salusin-*β* is more closely related to cardiovascular diseases. Salusin-*β* is expressed abundantly in peripheral vascular tissue, including VECs and VSMCs [21–23]. Even though studies have found that salusin-*β* might be a ligand of the mas-like G protein-coupled receptor or *β*-chain of ATP synthase [24–26], its exact receptor has not yet been discovered or confirmed. It has been reported that salusin-*β* regulates blood pressure [27], activates sympathetic outflow [28], and promotes the proliferation, migration, and foam cell formation of VSMCs [29–31]. More importantly, salusin-*β* is closely related to vascular endothelial function [29, 32]. Studies have found that salusin-*β* is involved in endothelial injury and dysfunction in diabetes mellitus [30, 33]. Recently, we also found that salusin-*β* plays important roles in regulating endothelial dysfunction in spontaneously hypertensive rats [34, 35]. It has been reported that salusin-*β* expression in the paraventricular nucleus (PVN) in aged spontaneously hypertensive rats with heart failure is dramatically increased, and salusin-*β* knockdown in the PVN attenuates sympathetic excitation and hypothalamic inflammation as well as cardiac and vascular dysfunction [36]. However, whether the salusin-*β* level is also increased and whether salusin-*β* contributes to endothelial dysfunction and impaired cardiovascular function in myocardial infarction-induced CHF are still unknown.

Therefore, the present study was designed to determine whether salusin-*β* plays roles in regulating endothelial dysfunction, cardiovascular remodeling, and cardiac function by silencing salusin-*β*, and to explore its downstream molecular mechanisms in three important small arteries, CA, PA, and MA, of CHF and sham-operated (Sham) rats.

## 2. Materials and Methods

The experiments were carried out on male Sprague-Dawley rats that were housed in a temperature- and humidity-controlled animal room on a 12 h–12 h light to dark cycle with free access to standard chow and tap water. The procedures were approved by the Nanjing Medical University Experimental Animal Care and complied with the Guide for the Care and Use of Laboratory Animals published by the US National Institutes of Health (NIH publication, 8th edition, 2011).

### 2.1. Model of CHF

The myocardial infarction-induced CHF model was established by coronary artery ligation, as described in our previous studies [37, 38]. Briefly, the left coronary artery of rats weighing approximately 180 g was ligated near the point at which it branched off from the aorta. Approximately 20% of the animals died, and death occurred mainly during the first day after ligation. The Sham rats were treated in the same way as the CHF rats, except their coronary arteries were not ligated. Six to eight weeks after coronary ligation, the degree of CHF was assessed. The defined criteria for CHF were an elevated left ventricular end-diastolic pressure (LVEDP) (>13 mmHg) and a 40% decrease in the maximum first derivative of left ventricular pressure (LV +dP/dt_max_).

### 2.2. Silencing of Salusin-*β*

CHF and Sham rats underwent tail intravenous injection of adenoviral vectors encoding control shRNA or salusin-*β* shRNA (2 × 10^11^ plaque forming units/mL, constructed by Genomeditech Co. Shanghai, China) to knockdown salusin-*β* as previously reported [27]. After 2 weeks, specific knockdown of salusin-*β* was verified by measuring salusin-*β* levels in plasma and protein expression in the CA, PA, and MA of rats by using Western blot.

### 2.3. Echocardiographic Assessment of Left Ventricular (LV) Function

Echocardiography was performed under ketamine (25 mg/kg, ip) sedation using an ultrasound system (Vevo 2100, Visual Sonics, Canada) and a 21 MHz probe to evaluate LV function after salusin-*β* knockdown as we previously described [37]. The following parameters were measured: left ventricular end-diastolic diameter and systolic diameter (LVEDD and LVESD, respectively), interventricular septal thickness in diastole and systole (IVSd and IVSs, respectively), and left ventricular posterior wall thickness in diastole and systole (LVPWd and LVPWs, respectively). Left ventricular fractional shortening (FS) and ejection fraction (EF) were calculated. All measures were averaged over four consecutive cardiac cycles.

### 2.4. Hemodynamic Parameters Measurement

Two weeks after control or salusin-*β*-shRNA application, rats were intraperitoneally anesthetized with urethane (800 mg/kg) and *α*-chloralose (40 mg/kg) after weighing. First, the right carotid artery was cannulated and connected to a pressure transducer (MLT0380, ADInstruments, Australia) to record continuous arterial blood pressure. The systolic arterial pressure (SAP), diastolic arterial pressure (DAP), pulse pressure (PP), mean arterial pressure (MAP), and heart rate (HR) were then calculated. Next, the catheter was pushed into the left ventricle from the right carotid artery to record LV pressure. The LV peak systolic pressure (LVSP), LVEDP, LV developed pressure (LVDP), and LV +dP/dt_max_ were calculated.

### 2.5. Isometric Tension Measurements in Arteries

Isometric tension of arteries was measured to evaluate vascular endothelial function as described in our previous report [39]. Briefly, after hemodynamic parameters were measured, the heart, lung, and mesentery were isolated from rats. Heart weight and infarct size were measured. Then, the third-order CA, PA, and MA were isolated and cut into 1-1.2 mm segments. Arterial rings (1 arterial ring/artery/rat was used) were mounted in a four-chambered myograph (620 M, DMT, Denmark) with 20 *μ*m wires and set at a resting tension of 0.1 g. After arterial ring contraction induced by prostaglandin F2*α* (PGF 2*α*), six doses of ACh (10^−9^~10^−4^ mol/L) were administered in a dose-dependent manner to induce vascular relaxation. The degree of relaxation is shown as a percentage of PGF 2*α*-induced contraction.

### 2.6. Treatment with L-NAME in CHF Rats

The nitric oxide synthase (NOS) inhibitor L-NAME (50 mg/kg/day) was given to rats by gavage for four weeks. The control group was given saline. At the start of the third week of L-NAME application, knockdown of salusin-*β* was performed.

### 2.7. Measurement of Protein Expression in Arteries by Western Blot

Third-order CA, PA, or MA samples were isolated from rats, flash-frozen in liquid nitrogen, and stored at -70°C. Then, the artery tissues were homogenized and centrifuged. The tissue homogenate from the artery was subjected to Western blot analysis for determination of protein levels. The protein concentration was measured using a protein assay kit (BCA, Pierce, USA), loaded onto an SDS-PAGE gel, and then transferred to a polyvinylidene fluoride membrane. The membranes were then probed overnight at 4°C with anti-salusin-*β* IgG (1 : 1000, Clound-Clone Corp, USA), endothelial NOS (eNOS) antibody (1 : 1000, Cell Signaling Technology, USA), phospho-eNOS Ser1177 antibody (1 : 1000, Cell Signaling Technology, USA), NOX-2 antibody (1 : 1000, Abcam, USA), NOX-4 antibody (1 : 1000, Proteintech Inc., Wuhan, China), or *β*-actin antibody (1 : 5000, Abways Technology Inc., Shanghai, China) followed by incubation with horseradish peroxidase-conjugated goat anti-rabbit IgG (1 : 5000, Immunology Consultants Lab, Portland, OR, USA). Protein loading was controlled by probing all blots with *β*-actin antibody. The bands were visualized by an enhanced chemiluminescence ECL system (Pierce Chemical, Rockford, IL, USA). eNOS activity was evaluated by the ratio of phosphorylation of eNOS to total eNOS (p-eNOS/T-eNOS).

### 2.8. Measurement of Salusin-*β*, Leptin, Visfatin, eNOS, NO Levels, and eNOS Activity by ELISA

Commercial ELISA kits were used for the measurement of the plasma salusin-*β* (Cloud-clone corp, Wuhan, China), leptin (Hui Jia Biotechnology, Xiamen, China), visfatin (Hui Jia Biotechnology, Xiamen, China), and eNOS levels of arteries (Yi Fei Xue Biotechnology, Nanjing, China) according to the manufacturer's descriptions. A nitrate/nitrite colorimetric assay kit (Cayman Chemical Co., Ann Arbor, MI, USA) was used to evaluate NO production in arteries based on the detection of the concentration of its stable metabolites nitrate and nitrite. The measurement of eNOS activity by ELISA was performed using a Nitric Oxide Synthase Assay Kit (Beyotime Biotech Inc., Nanjing, China) by assessing the ability of conversion of L-arginine to NO.

### 2.9. Measurement of NAD(P)H Oxidase Activity and Reactive Oxygen Species (ROS) Levels

NAD(P)H oxidase activity and ROS levels were measured using the enhanced lucigenin-derived chemiluminescence method as we previously reported [27, 40–42]. Briefly, the light emissions produced by the reactions between lucigenin (5 *μ*M) and the ROS in tissue homogenate supernatant were measured by a luminometer (20/20n, Turner, CA, USA) once every minute for 10 minutes to measure ROS levels. To measure NAD(P)H oxidase activity, NAD(P)H (100 *μ*M) was first added to the medium as a substrate to react with NAD(P)H oxidase to generate ROS before the reactions between lucigenin and ROS were detected by a luminometer. The values representing NAD(P)H oxidase activity and ROS levels are expressed as the mean light unit (MLU) per minute per milligram of protein.

### 2.10. Evaluation of Vascular Remodeling

Third-order CA, PA, and MA isolated from rats were embedded in paraffin, cut into 5-*μ*m thick cross-sections, and stained with hematoxylin-eosin (HE). The structural changes of these arteries were observed with a light microscope. The media thickness, lumen diameter, and media thickness/lumen diameter were measured and used as indexes of vascular remodeling [27].

### 2.11. Evaluation of Left Ventricular Remodeling and Microvascular Density

Perivascular fibrosis in the intramuscular arteries and arterioles and myocardium fibrosis were evaluated in Masson's trichrome-stained sections under high and low power microscope as previously reported [27, 43]. Myocyte cross-sectional area was determined in the left ventricular lateral-midfree wall, including epicardial and endocardial portions, in HE-stained sections [44]. Immunohistochemistry staining of cardiomyocytes with dystrophin antibody (1 : 200, Proteintech Inc., Wuhan, China) was also performed to observe the change in cardiomyocyte morphology of rats. Paraffin sections of the myocardial infarct border and remote zone (apex of heart) were immunohistochemically stained with an endothelial marker CD31 antibody (1 : 500, Servicebio Inc., Wuhan, China) to show capillary and arteriolar density in the myocardial tissue [45].

### 2.12. Cell Experiments

Human pulmonary arterial endothelial cells (HPAECs) (ScienCell Research Laboratories, Carlsbad, CA, USA) were cultured in DMEM containing 10% FBS, 1% penicillin and streptomycin, and 1% growth factor at 37°C in an incubator containing 95% air and 5% CO_2_. Tumor necrosis factor-*α* (TNF-*α*) (10 *μ*g/mL) was added to DMEM for 24 h to stimulate cells to mimic the similar inflammatory responses produced in heart failure. Then, HPAECs were transfected with adenovirus-mediated shRNA against salusin-*β* or control shRNA (MOI = 100) for 48 h to silence salusin-*β* in vitro. Then, the eNOS activity, NO level, NAD(P)H oxidase activity, and ROS level of the cells were measured.

### 2.13. Chemicals

Salusin-*β* was obtained from Bachem (Bubendorf, Switzerland). Prostaglandin F2*α* (PGF 2*α*), acetylcholine (ACh), N′-nitro-L-arginine-methyl ester hydrochloride (L-NAME), and TNF-*α* were purchased from Sigma Chemical Co. (St. Louis, MO, USA). The chemicals were dissolved in normal saline.

### 2.14. Statistical Analysis

Data are expressed as the mean ± S.E. The Kolmogorov-Smirnov test and Shapiro-Wilk test were used to measure the normal distribution of values. Student's unpaired *t*-test was used for comparisons between two groups. One-way or two-way ANOVA was used, followed by the Bonferroni test for posthoc analysis when multiple comparisons were made. *P* < 0.05 was considered statistically significant.

## 3. Results

### 3.1. Effects of Salusin-*β* Knockdown on Anatomy and Hemodynamics

The body weight (BW), heart weight (HW), infarct area, and baseline hemodynamic arguments were measured after two weeks of cont-shRNA or salusin-*β*-shRNA application in CHF and Sham rats. Although the BW was not significantly different between CHF and Sham rats, the HW and the HW/BW were increased in CHF rats, which suggested the occurrence of myocardial hypertrophy. Knockdown of salusin-*β* decreased the infarct size of the LV in CHF rats. Compared with the Sham rats, the SAP, PP, LVSP, LVDP, and LV +dP/dt_max_ decreased, while LVEDP increased significantly in CHF rats, which was consistent with our previous study findings [46]. These abnormal hemodynamic parameters in CHF were improved by salusin-*β* knockdown (Table 1).

### 3.2. Effects of Salusin-*β* Knockdown on Echocardiography

Compared to Sham rats, the LVEDD, LVESD, LV mass, and LV mass/BW were obviously increased, while EF, FS, and LVPWs were significantly decreased in CHF rats. More importantly, these abnormal parameters, except LV mass and LV mass/BW, of CHF rats were improved by salusin-*β* knockdown (Table 2).

### 3.3. Effects of Salusin-*β* Knockdown on Salusin-*β* Levels in Plasma and Protein Expression in Arteries

**Figure 1:**
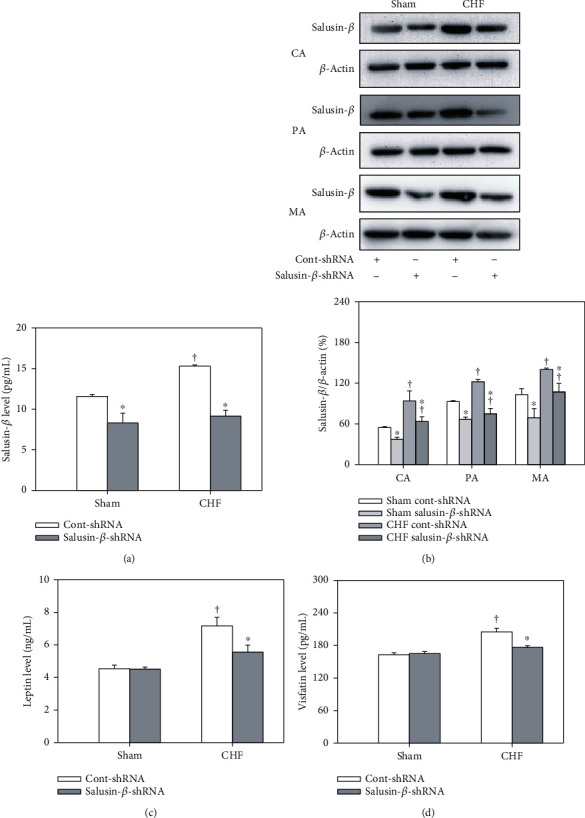
The salusin-*β* level in plasma (a), salusin-*β* protein expression of CA, PA, and MA (b), leptin (c), and visfatin levels (d) of plasma in Sham and CHF rats with salusin-*β* knockdown. Values are mean ± SE. ^∗^*P* < 0.05 compared with Cont-shRNA, ^†^*P* < 0.05 compared with Sham. *n* = 6 for each group.

The content of salusin-*β* in the plasma (Figure 1(a)) and salusin-*β* protein expression in the CA, PA, and MA (Figure 1(b)) of CHF rats were significantly higher than those of Sham rats. After tail intravenous injection of adenoviral vectors encoding salusin-*β* shRNA to knockdown salusin-*β*, the salusin-*β* levels in the plasma and salusin-*β* protein expression in arteries were decreased considerably in both CHF and Sham rats.

### 3.4. Effects of Salusin-*β* Knockdown on Plasma Leptin and Visfatin Levels

Both the leptin (Figure 1(c)) and visfatin levels (Figure 1(d)) in plasma were increased in CHF rats compared with Sham rats and were restored by salusin-*β*-shRNA intervention in CHF rats.

### 3.5. Effects of Salusin-*β* Knockdown on Endothelium-Dependent Vascular Relaxation

**Figure 2:**
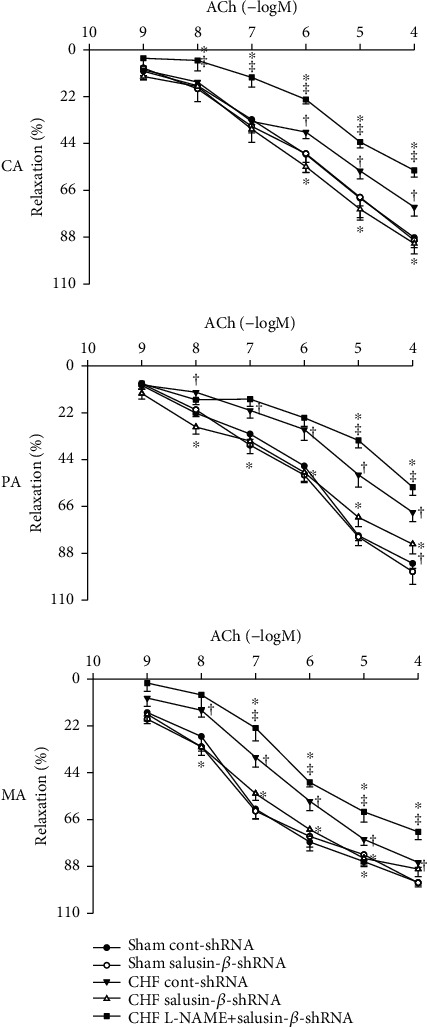
ACh-induced dose-dependent relaxation of CA, PA, and MA in Sham and CHF rats. Values are mean ± SE. ^∗^*P* < 0.05 compared with Cont-shRNA, ^†^*P* < 0.05 compared with Sham, ^‡^*P* < 0.05 compared with CHF salusin-*β*-shRNA. *n* = 6 for each group.

ACh-induced dose-dependent relaxations in CA, PA, and MA were attenuated in CHF rats compared with Sham rats and were ameliorated significantly by silencing salusin-*β*. Silencing of salusin-*β* had no significant effect on endothelium-dependent vascular relaxation in Sham rats (Figure 2).

### 3.6. Effects of L-NAME on Vascular Relaxation in Response to Salusin-*β* Knockdown

Pretreatment with the NOS inhibitor L-NAME in CHF rats significantly inhibited the improved effects of salusin-*β* knockdown on endothelium-dependent vascular relaxation (Figure 2).

### 3.7. Effects of Salusin-*β* Knockdown on eNOS Activity and Protein Expression and NO Levels in Arteries

**Figure 3:**
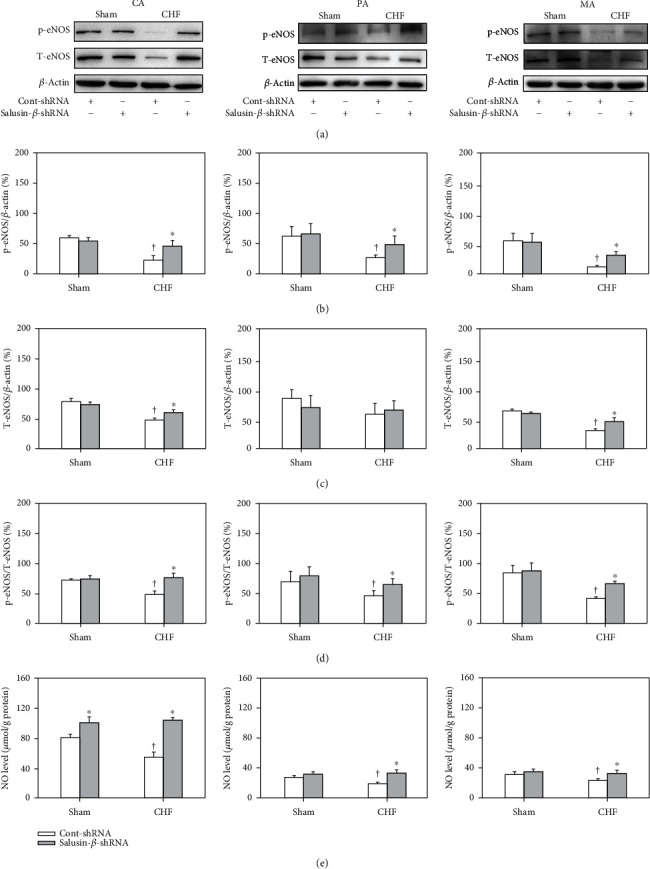
The protein expression of p-eNOS (a, b), T-eNOS (a, c), and p-eNOS/T-eNOS (d), as well as NO level (e) of CA, PA, and MA in Sham and CHF rats. Values are mean ± SE. ^∗^*P* < 0.05 compared with Cont-shRNA, ^†^*P* < 0.05 compared with Sham. *n* = 6 for each group.

Compared with Sham rats, the p-eNOS protein expression (Figures 3(a) and 3(b)) of CA, PA, and MA in CHF rats was decreased, and the T-eNOS protein expression (Figures 3(a) and 3(c)) of CA and MA in CHF rats was also decreased. eNOS activity was evaluated by p-eNOS/T-eNOS (Figure 3(d)), which was also significantly decreased in these three arteries of CHF rats. These results indicated that both the eNOS amount and activity of arteries were decreased in CHF rats. Consistent with this, NO levels (Figure 3(e)) in these three arteries of CHF rats were also much lower than those of Sham rats. They were all redressed by salusin-*β* knockdown. However, silencing salusin-*β* had no significant effect on eNOS activity and protein expression or NO levels in arteries, except CA, of Sham rats (Figure 3).

### 3.8. Effects of Salusin-*β* Knockdown on NAD(P)H Oxidase Activity and ROS Levels in Arteries

**Figure 4:**
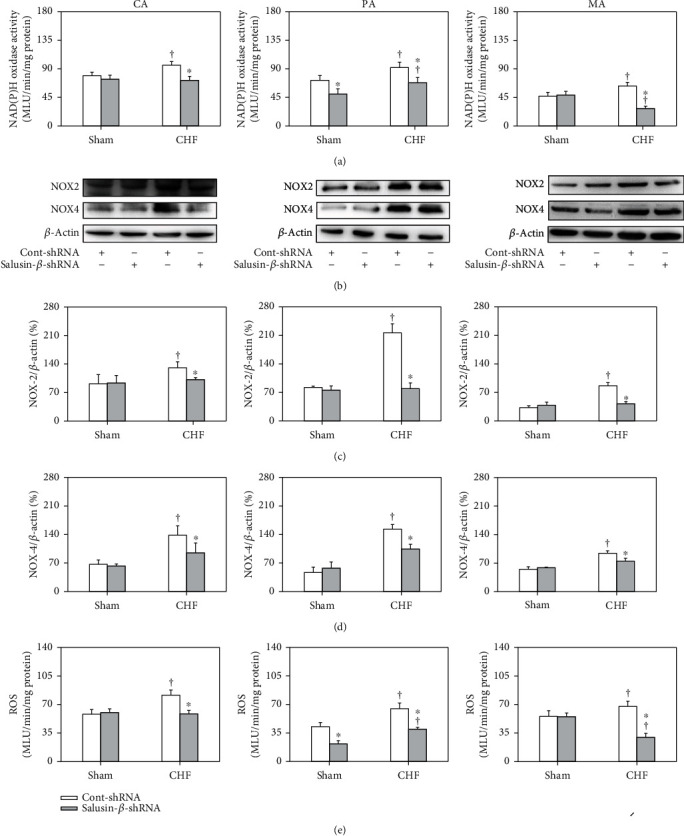
NAD(P)H oxidase activity (a), NOX-2 (b, c), and NOX-4 protein expression (b, d), as well as ROS levels (e), of CA, PA, and MA in Sham and CHF rats. Values are mean ± SE. ^∗^*P* < 0.05 compared with Cont-shRNA, ^†^*P* < 0.05 compared with Sham. *n* = 6 for each group.

In CHF rats, the NAD(P)H oxidase activity (Figure 4(a)) and NAD(P)H oxidase subunit NOX-2 (Figures 4(b) and 4(c)) and NOX-4 (Figures 4(b) and 4(d)) protein expression of CA, PA, and MA were increased significantly. The level of ROS (Figure 4(e)) in arteries in CHF rats was higher than that of Sham rats. They were all corrected by salusin-*β* knockdown in CHF rats. However, silencing salusin-*β* had no significant effect on NAD(P)H oxidase activity or NOX-2 and NOX-4 protein expression or ROS levels in the arteries of Sham rats (Figure 4).

### 3.9. Effects of L-NAME on NO and ROS Responses to Salusin-*β* Knockdown

The application of L-NAME to CHF rats significantly inhibited the elevating effects of salusin-*β* knockdown on NO levels and the depressing effects on NAD(P)H oxidase activity and ROS levels (Table 3).

### 3.10. Effects of Salusin-*β* Knockdown on Vascular Remodeling

**Figure 5:**
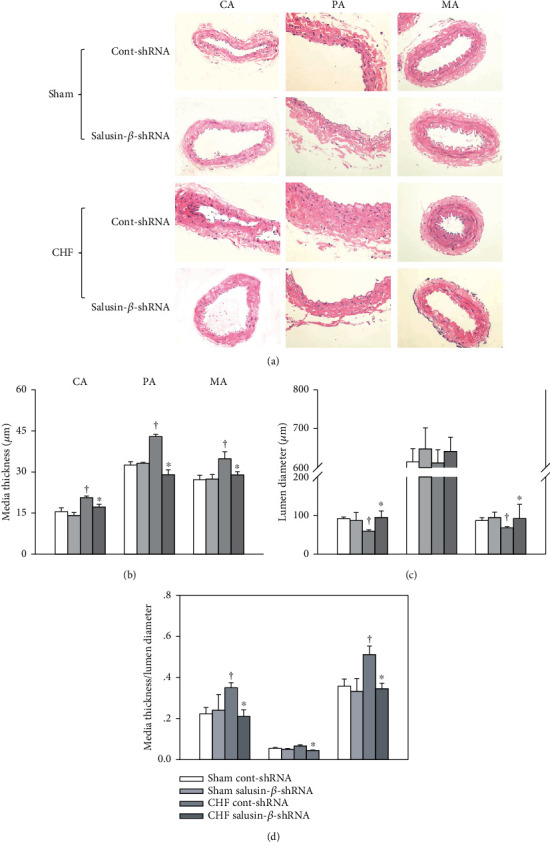
The media thickness (a, b), lumen diameter (a, c), and media thickness/lumen diameter (d) of CA, PA, and MA in Sham and CHF rats. Values are mean ± SE. ^∗^*P* < 0.05 compared with Cont-shRNA, ^†^*P* < 0.05 compared with Sham. *n* = 6 for each group.

The lumen diameters of the CA, PA, and MA (Figures 5(a) and 5(c)) of CHF rats were reduced, while the media thickness (Figures 5(a) and 5(b)) and the ratio of media thickness to lumen diameter (Figure 5(d)) were increased compared with those of Sham rats, which indicated the occurrence of vascular remodeling. Silencing of salusin-*β* in CHF rats increased the lumen diameter and decreased the media thickness and the ratio of media thickness to lumen diameter of CA, PA, and MA, suggesting that vascular remodeling was improved by salusin-*β* knockdown (Figure 5).

### 3.11. Effects of Salusin-*β* Knockdown on Left Ventricular Remodeling and Microvascular Density

**Figure 6:**
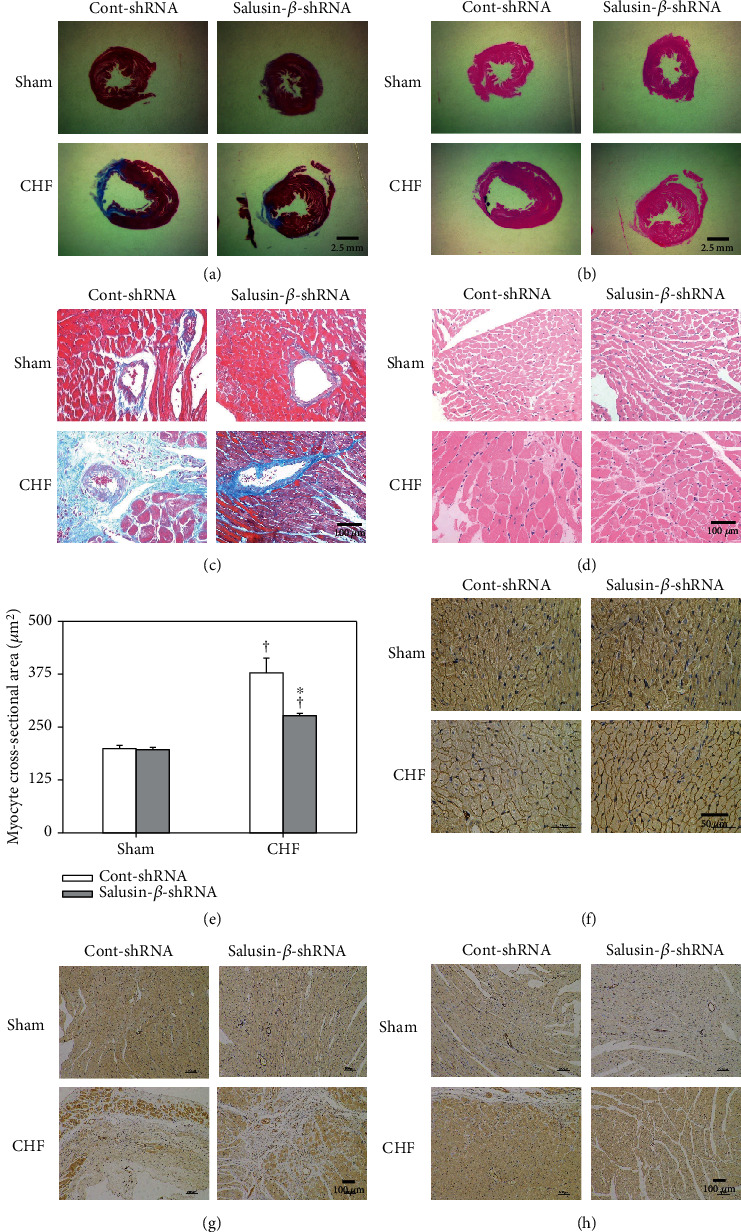
Effects of salusin-*β* knockdown on left ventricular remodeling and microvascular density. Sections of myocardium with Masson's stain under low- (a) and high-power microscope (c) showing fibrosis. Sections of myocardium with HE staining under low- (b) and high-power microscopy (d) and dystrophin staining (f) showing the size of cardiomyocytes. Bar graph showing the quantitative analysis of the cross-sectional area of cardiomyocytes (e). Sections of myocardial infarct border (g) and remote zone (apex of heart) (h) with endothelial marker CD31 immunohistochemistry staining showing the microvascular density. Values are mean ± SE. ^∗^*P* < 0.05 compared with Cont-shRNA, ^†^*P* < 0.05 compared with Sham. *n* = 6 for each group.

Severe perivascular and myocardium fibrosis of sections of myocardium with Masson's stain (Figures 6(a) and 6(c)), cardiomyocyte hypertrophy with HE stain (Figures 6(b) and 6(d)), and increased cross-sectional area of cardiomyocytes (Figure 6(e)) of left ventricular were observed in CHF. Morphometric dystrophin staining of cardiomyocytes also showed the occurrence of cardiomyocyte hypertrophy in CHF rats (Figure 6(f)). They were all blunted by salusin-*β* knockdown. Furthermore, the capillary and arteriolar density was increased significantly in the infarcted area after salusin-*β* silencing in CHF rats (Figure 6(g)), which might suggest the occurrence of neovascularization in the infarcted area of CHF rats after salusin-*β* knockdown. However, there was no significant difference in microvascular density in the noninfarcted area (apex of heart) (Figure 6(h)) between Sham and CHF rats treated with either Cont-shRNA or salusin-*β*-shRNA.

### 3.12. Effects of Salusin-*β* Knockdown on NO and ROS of Cells

**Figure 7:**
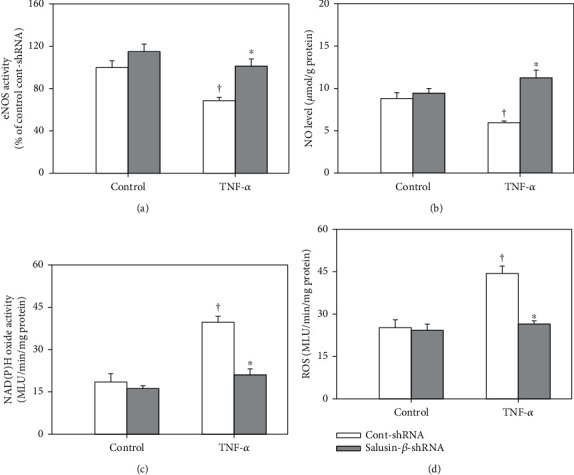
The effects of salusin-*β* knockdown on eNOS activity (a), NO levels (b), NAD(P)H oxidase activity (c), and ROS levels (d) in HPAECs stimulated by TNF-*α* (10 ng/mL). Values are mean ± SE. ^∗^*P* < 0.05 compared with Cont-shRNA, ^†^*P* < 0.05 compared with control. *n* = 6 for each group.

After the application of TNF-*α* to HPAECs, the eNOS activity (Figure 7(a)) and NO level (Figure 7(b)) in the cells were decreased, while the activity of NAD(P)H oxidase (Figure 7(c)) and the level of ROS (Figure 7(d)) were increased, which was consistent with the situation that occurred in CHF. The changes induced by TNF-*α* were also reversed by silencing salusin-*β* gene expression in cells.

### 3.13. Effects of Salusin-*β* Knockdown on NAD(P)H Oxidase Activity and ROS and NO Levels in Cardiac Tissues

We found that there was no significant difference of salusin-*β* protein expression in cardiac tissues between Sham and CHF rats. Compared with Sham rats, the NAD(P)H oxidase activity and ROS level in cardiac tissues in CHF rats were increased, and NO levels were decreased. However, they were not influenced by tail intravenous injection of salusin-*β* shRNA (Supplemental Figure S1). These results indicated that salusin-*β* in cardiomyocytes does not play a direct role in regulating the NO and ROS signaling pathways in rats, which excluded the direct effect of salusin-*β* knockdown on cardiomyocytes.

### 3.14. The Salusin-*α* Level in Sham and CHF Rats

There were no significant differences in the plasma level of salusin-*α* or its protein expression in the three arteries, MA, CA, and PA, or cardiac tissues between Sham and CHF rats (Supplemental Figure S2). We speculated that salusin-*α* might not be involved in the pathogenesis of CHF.

## 4. Discussion

Endothelium-dependent diastolic dysfunction is closely related to the occurrence and development of CHF and could trigger deteriorating events, such as cardiovascular remodeling, in CHF patients. The CA, PA, and MA are three essential arteries related to endothelial dysfunction in CHF. The major new findings of the present study were (1) The levels of salusin-*β*, leptin, and visfatin in plasma and salusin-*β* protein expressions of CA, PA, and MA of CHF were much higher than that of Sham rats, which were decreased significantly by salusin-*β* knockdown; (2) endothelium-dependent vascular relaxation was attenuated in CHF which was improved by salusin-*β* knockdown; (3) the eNOS activity and protein expressions as well as NO level in the three arteries of CHF were lower than those of Sham rats, while NAD(P)H oxidase activity, NOX-2 and NOX-4 protein expressions, and ROS level of arteries were higher than those of the Sham rats. After salusin-*β* knockdown, these abnormalities were substantially improved; (4) the improved effects of salusin-*β* knockdown on ACh-induced vascular relaxation, NO level, NAD(P)H oxidase activity, and ROS level of arteries of CHF were inhibited by L-NAME pretreatment; (5) the interference of salusin-*β* gene expression in CHF rats improved cardiac function, vascular remodeling, and left ventricular remodeling and promotes the angiogenesis in infarct zone of heart; (6) silencing of the salusin-*β* gene expression also reversed the depressed eNOS activity and NO level as well as the increased NAD(P)H oxidase activity and the ROS level of HPAECs stimulated by TNF-*α*. These results indicated that salusin-*β* is closely related to endothelial dysfunction and vascular and ventricular remodeling in chronic heart failure. Knockdown of salusin-*β* contributes to the improvement of endothelial dysfunction, cardiac function, and cardiovascular remodeling in CHF by inhibiting vascular NAD(P)H oxidase-derived ROS generation, activating eNOS and increasing NO production.

Salusins are vasoactive peptides, including salusin-*α* (28 amino acids) and salusin-*β* (20 amino acids) [18]. Salusin-*β* is more closely related to cardiovascular diseases than salusin-*α* [32, 47]. Salusin-*β* is expressed in the hypothalamus, posterior pituitary gland, gastrointestinal tract, immune system, endocrine system, and peripheral vascular tissue, and it has rich expression in VECs and VSMCs [48–50]. The plasma salusin-*β* levels in patients with diabetes mellitus [47], coronary artery disease [51, 52], and hypertension [36, 53] are distinctly higher than those in healthy controls. It has been reported that salusin-*β* regulates blood pressure [27], activates sympathetic outflow [28], and promotes the proliferation, migration, and foam cell formation of VSMCs [29–31]. More importantly, salusin-*β* is closely related to vascular endothelial function [29, 32]. Salusin-*β* causes endothelial injury and dysfunction in diabetes mellitus [47, 54, 55] and promotes the inflammatory response of human umbilical vein endothelial cells through NF-*κ*B signaling [47, 56]. However, whether the salusin-*β* level in myocardial infarction-induced CHF is also increased and whether salusin-*β* is involved in endothelial dysfunction and impaired cardiovascular function in CHF are still unknown. The present study found that both the plasma salusin-*β* level and its protein expressions in the CA, PA, and MA of CHF rats were much higher than those of the Sham rats, which indicated that the activity of salusin-*β* in CHF was increased. However, there were no significant differences in the salusin-*α* plasma level and its protein expressions in MA, CA, and PA and cardiac tissues between Sham and CHF rats, which suggested that salusin-*α* might not be involved in the pathogenesis of CHF.

It has been reported that plasma leptin and visfatin levels were increased in patients with myocardial infarction or other cardiovascular diseases [57, 58]. High levels of leptin increase oxidative stress in endothelial cells, reduce vasodilatation, and contribute to obesity-related hypertension [59]. Visfatin causes vascular endothelial dysfunction, inhibits the production of NO and vasodilatation, and promotes the proliferation of vascular smooth muscle cells [60]. Leptin and visfatin have been proposed as clinical markers of atherosclerosis, endothelial dysfunction, and vascular injury in cardiovascular disease. In this study, the leptin and visfatin levels in plasma were increased, while ACh-induced endothelium-dependent vascular relaxation was significantly attenuated in CHF, which suggested the occurrence of endothelial dysfunction in CHF rats. Salusin-*β* knockdown by shRNA improved endothelium-dependent vascular relaxation and decreased the plasma leptin and visfatin levels in CHF. These results indicated that salusin-*β* is a critical regulator of endothelial function in CHF, and the elevated activity of salusin-*β* in the circulatory system in CHF might be an important cause of endothelial dysfunction in CHF.

It has been reported that salusin-*β* stimulates the production of NAD(P)H oxidase-derived ROS in human umbilical vein endothelial cells [61–64]. Through the oxidative stress-related signaling pathway, salusin-*β* stimulates the migration of VSMCs and intimal hyperplasia after vascular injury [47, 65]. Salusin-*β* promotes the formation of foam cells and monocyte adhesion in atherosclerosis by stimulating the production of ROS [30, 31]. Salusin-*β* enhances oxidative stress and inflammation in diabetic cardiomyopathy, and knockdown of salusin-*β* improves cardiac dysfunction and decreases oxidative stress and inflammation in diabetic cardiomyopathy [47]. In this study, it was found that both NAD(P)H oxidase activity and the protein expression of its subunits NOX-2 and NOX-4 in the CA, PA, and MA of CHF rats were increased. In addition, the ROS levels in the arteries of CHF rats were much higher than those of Sham rats. Silencing salusin-*β* decreased the elevated NAD(P)H oxidase activity, NOX-2 and NOX-4 protein expression, and ROS levels in the arteries of CHF rats, which suggested that the activation of NAD(P)H oxidase and elevated ROS production played important roles in mediating the effects of salusin-*β*. In addition, we also found that the eNOS activity and protein expression and NO level in the three arteries of CHF rats were lower than those of Sham rats and were substantially improved by knockdown of salusin-*β*. The effects of salusin-*β* knockdown on ACh-induced vascular relaxation, NO levels, NAD(P)H oxidase activity, and ROS levels in CHF arteries were markedly inhibited by pretreatment with the NOS inhibitor L-NAME. Although the relationship between salusin-*β* and NO is rarely reported at present, the above results suggested that lowering salusin-*β* levels in circulation alleviated oxidative stress and improved damaged eNOS-NO production in CHF, which might be the major reason for the improvement of endothelial dysfunction by silencing salusin-*β* expression.

CHF is a chronic inflammatory response, and TNF-*α* plays an important role in its pathogenesis [66]. Studies have found that the levels of TNF-*α* in the peripheral circulation or heart tissues of patients with CHF are significantly increased [67, 68]. In the present study, we used TNF-*α* to stimulate HPAECs to simulate the injury of endothelial cells suffering from chronic heart failure and found that the eNOS activity and NO levels of the cells were decreased, while NAD(P)H oxidase activity and ROS levels were increased, which were also reversed by cell transfection with salusin-*β* shRNA. Therefore, our in vitro cell experiments again verified the results found in animal experiments.

In this study, echocardiography showed that the EF and FS were markedly decreased, while the LVEDD, LVESD, LV mass, and LV mass/BW were obviously increased in CHF rats, suggesting the significant reduction of cardiac function and the occurrence of left ventricular compensatory myocardial hypertrophy in CHF rats. Masson's stain, HE staining, and dystrophin staining further revealed severe perivascular and myocardial fibrosis and cardiomyocyte hypertrophy in the LV myocardium of CHF. In addition, the lumen diameter of the CA, PA, and MA in CHF decreased, while the media thickness and media thickness/lumen diameter were significantly increased, suggesting the occurrence of vascular remodeling in CHF. The interference of salusin-*β* gene expression in CHF rats improved cardiac function, vascular remodeling, and left ventricular remodeling and simultaneously induced neovascularization in the infarcted region of CHF rats, which might be the important reason for the decrease in the infarcted area. The mechanisms of these improvements in CHF are still not clear, but some studies have indicated that NO released from endothelial cells might be an inhibitor of vascular remodeling [69, 70]. Therefore, we speculated that the improvement in vascular remodeling might be due to the improvement in endothelial function and the increase in eNOS-NO production in arteries induced by salusin-*β* silencing. Then, improved vascular relaxation of CA in CHF increases the blood supply to the heart and relieves myocardial ischemia; improved MA relaxation decreases total peripheral resistance and, more importantly, decreases cardiac afterload, which all contribute to the subsequent improvement of left ventricular remodeling and cardiac function. In addition, improved PA relaxation in CHF decreased pulmonary vascular resistance and the incidence of pulmonary hypertension due to left heart disease.

In addition, we found that there were no significant differences in salusin-*β* protein expression in cardiac tissues between Sham and CHF rats. Compared with Sham rats, the NAD(P)H oxidase activity and ROS level in cardiac tissues in CHF rats were increased, and NO level were decreased, which suggested the increased oxidative stress and impaired NO bioavailability also occurred in hearts of CHF rats. However, NAD(P)H activity, ROS, and NO levels were not influenced by tail intravenous injection of salusin-*β* shRNA. These results indicated that salusin-*β* in cardiomyocytes might not play direct roles in regulating the NO and ROS signaling pathways in CHF rats, which excluded the direct effect of salusin-*β* knockdown on cardiomyocytes.

In conclusion, the present study indicates that salusin-*β* in the circulatory system is closely related to endothelial dysfunction, cardiovascular remodeling, and cardiac dysfunction in CHF. Silencing salusin-*β* contributes to the improvement of endothelial function, cardiac function, and cardiovascular remodeling in CHF by inhibiting NAD(P)H oxidase-derived ROS generation and activating eNOS and NO production, which provides a new strategy and target for the treatment of chronic heart failure in the future.

## Figures and Tables

**Table 1:** BW, HW, infracted area, and baseline hemodynamics in a representative group of CHF and Sham rats after two weeks of Cont-shRNA or Salusin-*β*-shRNA application.

	Sham		CHF	
	Cont	Salusin-*β*	Cont	Salusin-*β*
BW (g)	424 ± 5.9	408 ± 21.1	415 ± 7.3	418 ± 21.6
HW (g)	1.3 ± 0.1	1.2 ± 0.1	1.9 ± 0.0^†^	1.8 ± 0.1^†^
HW/BW (g/kg)	3.0 ± 0.1	2.9 ± 0.1	4.6 ± 0.1^†^	4.3 ± 0.2^†^
Infarct size (% LV area)	0	0	43.1 ± 1.0^†^	30.4 ± 1.0^∗^^†^
SAP (mmHg)	142.5 ± 4.2	139.8 ± 3.5	112.5 ± 3.2^†^	122.4 ± 3.6^∗^^†^
DAP (mmHg)	103.5 ± 5.1	102.5 ± 6.7	95.6 ± 7.2	86.7 ± 6.2
PP (mmHg)	39.0 ± 7.2	37.2 ± 6.9	17.0 ± 2.9^†^	35.7 ± 3.0^∗^
MAP (mmHg)	119.5 ± 6.7	116.2 ± 6.4	102.3 ± 5.2^†^	97.5 ± 6.1^†^
HR (beats/min)	386.9 ± 26.2	335.9 ± 20.6	358.7 ± 38.2	375.9 ± 26.1
LVSP (mmHg)	137.9 ± 6.1	129.7 ± 7.3	97.9 ± 3.3^†^	109.8 ± 4.6^∗^^†^
LVEDP (mmHg)	2.5 ± 1.7	1.9 ± 1.4	13.5 ± 0.7^†^	10.3 ± 0.5^∗^^†^
LVDP (mmHg)	135.3 ± 6.7	127.7 ± 7.0	84.5 ± 5.2^†^	99.5 ± 4.5^∗^^†^
LV +dP/dt_max_ (mmHg/sec)	3669.8 ± 396.0	3725.9 ± 423.0	1981.7 ± 133.3^†^	3389.6 ± 333.9^∗^

BW: body weight; HW: heart weight; SAP: systolic arterial pressure; DAP: diastolic arterial pressure; PP: pulse pressure; MAP: mean arterial pressure; HR: heart rate; LV: left ventricle; LVSP: left ventricle peak systolic pressure; LVEDP: left ventricle end-diastolic pressure; LVDP: left ventricle developed pressure; LV +dP/dt_max_: maximum of the first derivative of left ventricle pressure. Data are given as mean ± SE. ^∗^*P* < 0.05 vs. Cont-shRNA. ^†^*P* < 0.05 vs. Sham. *n* = 6 for each group.

**Table 2:** Echocardiographic data of the left ventricle in a representative group of Sham and CHF rats after two weeks of Cont-shRNA or salusin-*β*-shRNA application.

	Sham		CHF	
	Cont	Salusin-*β*	Cont	Salusin-*β*
LVEDD, mm	7.12 ± 0.23	6.76 ± 0.43	11.36 ± 0.21^†^	9.24 ± 0.28^∗^^†^
LVESD, mm	4.65 ± 0.13	3.49 ± 0.30	9.27 ± 0.40^†^	7.86 ± 0.25^∗^^†^
IVSd, mm	1.79 ± 0.05	1.70 ± 0.10	1.28 ± 0.13	1.40 ± 0.19
IVSs, mm	2.86 ± 0.13	2.93 ± 0.14	1.86 ± 0.26	1.76 ± 0.26
LVPWd, mm	1.86 ± 0.03	1.97 ± 0.12	1.63 ± 0.10	2.02 ± 0.07
LVPWs, mm	3.21 ± 0.11	3.08 ± 0.11	2.46 ± 0.30^†^	2.97 ± 0.08^∗^
FS, %	43.41 ± 1.24	48.54 ± 1.90	17.84 ± 1.02^†^	25.52 ± 2.01^∗^^†^
EF, %	72.60 ± 1.33	78.59 ± 1.83	34.73 ± 1.78^†^	47.55 ± 3.30^∗^^†^
LV mass (g)	1.19 ± 0.08	1.03 ± 0.17	1.55 ± 0.10^†^	1.53 ± 0.08^†^
LV mass/BW (mg/g)	2.81 ± 0.33	2.52 ± 0.47	3.73 ± 0.47^†^	3.66 ± 0.32^†^

LVEDD: left ventricular end-diastolic diameter; LVESD: left ventricular end-systolic diameter; IVSd: interventricular septal thickness in diastole; IVSs: interventricular septal thickness in systole; LVPWd: left ventricular posterior wall thickness in diastole; LVPWs: left ventricular posterior wall thickness in systole; FS: fractional shortening; EF: ejection fraction; LV: left ventricular; BW: body weight. Values are mean ± SE. ^∗^*P* < 0.05 vs. Cont-shRNA. ^†^*P* < 0.05 vs. Sham. *n* = 6 for each group.

**Table 3:** Influence of gavage treatment with saline and L-NAME on the NO level, NAD(P)H oxidase activity, and ROS level of CA, PA, and MA in CHF rats with Cont-shRNA or salusin-*β*-shRNA.

	Saline		L-NAME	
	Cont-shRNA	Salusin-*β*-shRNA	Cont-shRNA	Salusin-*β*-shRNA
*CA*				
NO level (*μ*mol/g protein)	54.6 ± 3.7	98.4 ± 5.4^∗^	26.6 ± 6.3^†^	39.0 ± 3.9^†^
NAD(P)H oxidase activity (MLU/min/mg protein)	96.1 ± 5.6	74.3 ± 2.3^∗^	151.1 ± 13.6^†^	165.8 ± 1.8^†^
ROS level (MLU/min/mg protein)	81.7 ± 4.5	63.1 ± 0.8^∗^	130.3 ± 9.7^†^	142.4 ± 2.5^†^
*PA*				
NO level (*μ*mol/g protein)	19.1 ± 2.2	30.3 ± 4.8^∗^	11.9 ± 1.0^†^	13.2 ± 1.9^†^
NAD(P)H oxidase activity (MLU/min/mg protein)	90.3 ± 4.2	63.0 ± 6.1^∗^	130.3 ± 7.7^†^	117.3 ± 5.5^†^
ROS level (MLU/min/mg protein)	65.1 ± 5.6	46.9 ± 4.3^∗^	96 ± 6.7^†^	103.6 ± 6.0^†^
*MA*				
NO level (*μ*mol/g protein)	23.3 ± 2.5	33.5 ± 3.3^∗^	10.1 ± 0.7^†^	11.9 ± 1.6^†^
NAD(P)H oxidase activity (MLU/min/mg protein)	63.3 ± 5.7	24.1 ± 5.9^∗^	103.3 ± 6.8^†^	98.0 ± 3.5^†^
ROS level (MLU/min/mg protein)	67.6 ± 6.4	21.0 ± 4.7^∗^	121.7 ± 7.4^†^	101.4 ± 6.1^†^

Values are expressed as mean ± SE. ^∗^*P* < 0.05 vs. Cont-shRNA, ^†^*P* < 0.05 vs. saline. *n* = 6 for each group.

## Data Availability

The data that support the findings of this study are available from the corresponding author upon reasonable request.

## Supplementary Materials

Supplementary Materials Supplemental figures associated with this article can be found in the Supplemental file.
