# Supplementary material for: Knockdown of Salusin-β Improves Cardiovascular Function in Myocardial Infarction-Induced Chronic Heart Failure Rats
Source: Oxid Med Cell Longev. 2021 Aug 10;2021:8896226. doi: 10.1155/2021/8896226 (PMC8373485; doi:10.1155/2021/8896226)
Supplement: Supplementary Materials — Supplemental figures associated with this article can be found in the Supplemental file. [file 8896226.f1.docx]

**Supplemental Figures for**

**Knockdown of Salusin-β Improves Cardiovascular Function in** **Myocardial Infarction-Induced Chronic Heart Failure Rats**

Yu Xu^1,2^, Yan Pan^1,2^, Xingxing Wang^1,2^, Aidong Chen^1,2^, Xinyu Tang^1,2^, Xuanxuan Liu^3^, Ying Han^1,2,*^

**
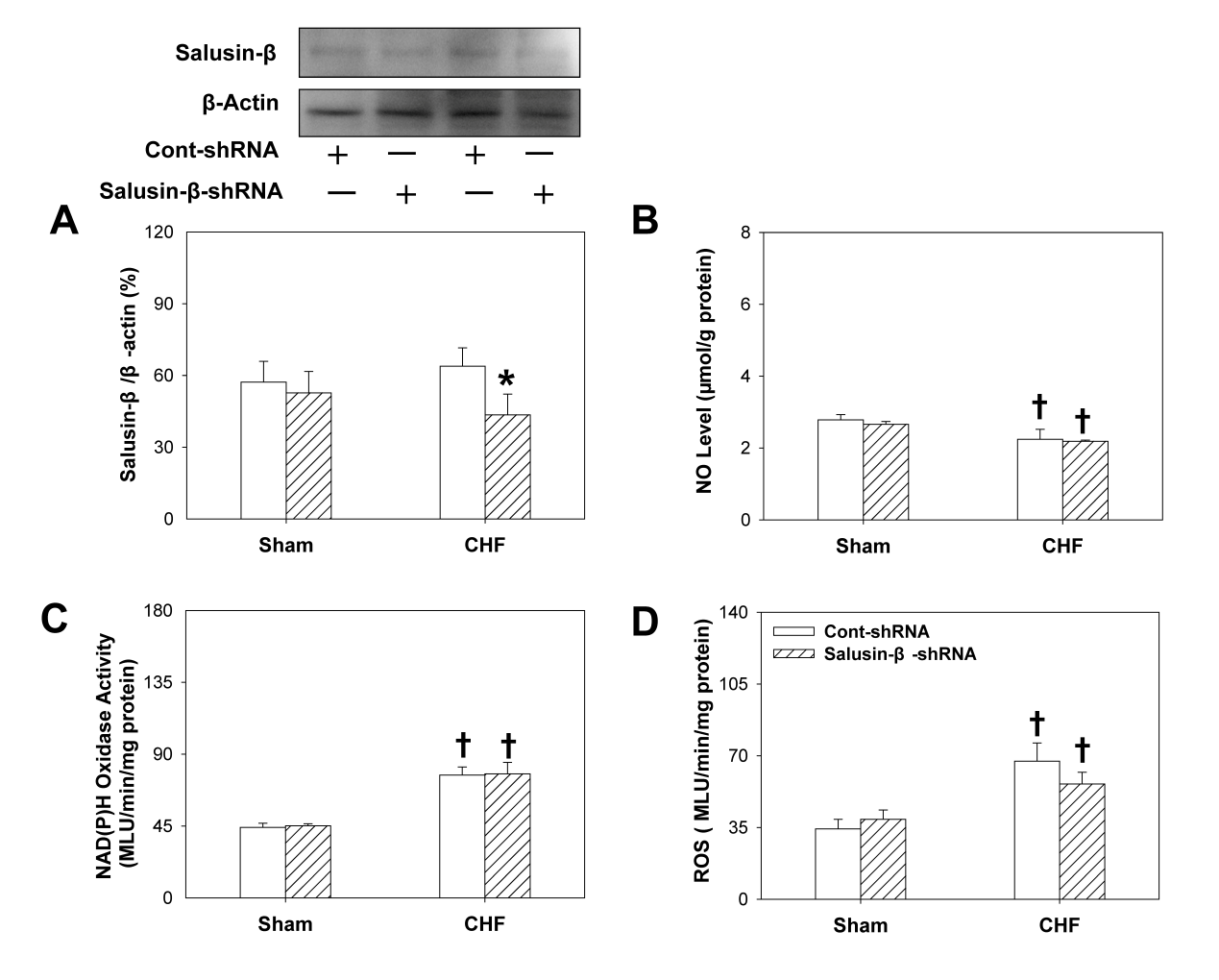
Supplemental Figure S1.** The effects of salusin-β knockdown on salusin-β protein expression (A), NO level (B), NAD(P)H oxidase activity (C), and ROS levels (D) of cardiac tissues in Sham and CHF rats. Values are mean ± SE. *P<0.05 compared with Cont-shRNA, †P<0.05 compared with Sham. n=6 for each group.


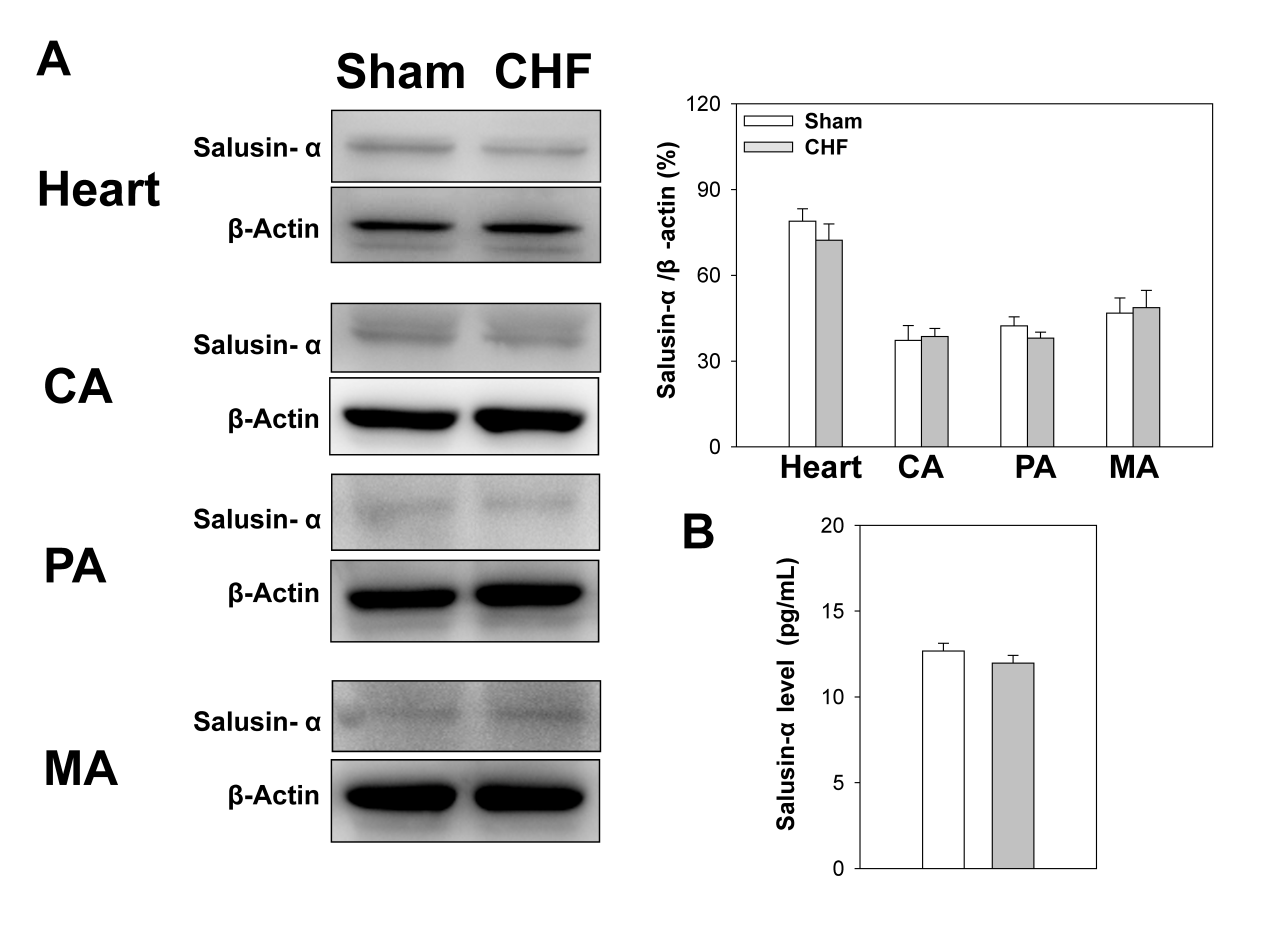


**Supplemental Figure S2.** The salusin-α protein expression of cardiac tissues, CA, PA and MA (A), and salusin-α level in plasma (B) in Sham and CHF rats. Values are mean ± SE. n=3 for each group in A, n=6 for each group in B.
